# The Payment of Hospital Staffs.—A Rejoinder to Dr. Heron

**Published:** 1910-08-27

**Authors:** Sydney Holland

**Affiliations:** London Hospital, Whitechapel, E.


### EDITOR'S LETTER-BOX.

### THE PAYMENT OF HOSPITAL STAFFS.—A REJOINDER TO DR. HERON.

*To the Editor of* The Hospital.

Dear Sir,—When I read Dr. Heron's letter expressing regret for having said, as he was reported to have said (but thinks he did not say), that a statement made by me "was one of the most disgraceful a man had ever made in public," I thought to myself, "Here is a gentleman—a man with good feeling enough to apologise for words spoken impromptu."

But the pleasure of reading his frank disavowal is considerably modified by what he now, on reflection, thinks fit to write of what I said. He writes that my words were insulting to medical men who undertake unpaid work in public hospitals, and unworthy of a man occupying my position in the hospital world. I will give my actual words. It is a remarkable fact, if these words were so insulting, that they passed without comment and without protest at the time, though there were many medical men present, and though Dr. Heron himself and six other medical men spoke after me! Now for the first time, two years and four months after I made the speech, am I held up as having said something unworthy of myself, and insulting to Dr. Heron's profession!

I was speaking in April 1908 at the United Kingdom Hospitals' Conference on the subject of payment by patients, and said:—

"Nobody would say that the staff of a hospital *gave* their services to the hospital for nothing. We are long past the day of thinking that it was not to the advantage of a medical man to be on the staff of a big hospital. Medical men belonged to a big hospital because they got a *quid pro quo* for their services. Any man who belonged to the staff of a hospital knew that it was an advertisement for him in his profession, that it was a step up in his profession, and that it enabled him to get patients."

I went on to say that medical men showed the greatest kindness to the patients, did a great deal more than they need do for them, and that they never spared themselves in trying to serve them; and I concluded this part of the argument by asking the Conference "to abolish the nonsense and hypocrisy in saying that medican men *gave* their services for nothing, because they did not."

Is this true? And if true, why insulting? Medical men do *not* join the staff of a hospital out of philanthropic motives, though this does not prevent their being philanthropic, as I gladly testify on every possible occasion.

Years ago, when I was an ambitious and struggling barrister, and when I was "devilling" for other men and doing their work for nothing, should I have been justified in thinking myself insulted if someone had said that I was not doing other men's work out of a kindly feeling for them, but was doing it simply to give solicitors the advantage of hearing my marvellous eloquence in the hope of beguiling the more innocent of them to give me employment? Surely not. The sad fact that they did not appreciate my powers does not alter my motive in doing this work for which my absent friends got paid, though Dr. Heron, in another part of his speech, is reported to have said that I must know lots of men who have never got any good from being on the staff of a hospital. They joined the staff just as I acted as "devil," in the hope of getting good for themselves by so doing. And if any have failed it has probably been their own fault as it was mine.

Yours faithfully,

London Hospital, SYDNEY HOLLAND.

Whitechapel, E., August 24, 1910.

